# Variation in Prolactin Is Related to Variation in Sexual Behavior and Contact Affiliation

**DOI:** 10.1371/journal.pone.0120650

**Published:** 2015-03-23

**Authors:** Charles T Snowdon, Toni E Ziegler

**Affiliations:** 1 Department of Psychology, University of Wisconsin, Madison, Wisconsin, United States of America; 2 Wisconsin National Primate Research Center, Madison, Wisconsin, United States of America; CNRS, FRANCE

## Abstract

Prolactin is associated with both maternal and paternal care and appears important in developing a bond between parent and infant. In contrast with oxytocin, another hormone important in infant care, there is scant information on the role of prolactin in maintaining adult heterosexual relationships. We present here the first results demonstrating a relationship between prolactin levels and sexual and contact affiliation behavior in a pair-bonded species. We studied cotton-top tamarins, a socially-monogamous, cooperatively-breeding primate. We measured chronic urinary prolactin levels over a four week period to include the entire female ovulatory cycle and correlated prolactin levels in males and females with simultaneous measures of contact affiliation and sexual behavior. Current mothers who were no longer nursing displayed lower amounts of sexual behavior and proximity than non-breeding females and also had marginally lower levels of prolactin. The prolactin levels of males and females were similar within pairs, and variation in prolactin levels for both sexes was explained both by the amount of sexual behavior and contact affiliation. The results parallel a previous study that compared oxytocin levels with sociosexual behavior in the same species, and supports the hypothesis that both prolactin and oxytocin are involved in pair-bonding as well as in infant care.

## Introduction

Despite the many parallels between prolactin and oxytocin in parental care and the interest in oxytocin in promoting pair bonding in monogamous biparental species [1], it is surprising that there are few papers on the role of prolactin in promoting pair-bonds. We have previously shown in the socially-monogamous, cooperatively-breeding cotton-top tamarin that peripheral oxytocin levels varied in both males and females and were correlated with sociosexual behavior (copulation, huddling and grooming) [2]. Within pairs, oxytocin levels were highly correlated between males and females. Variation in male oxytocin was explained best by variation in amount of sexual behavior observed whereas variation in female oxytocin was best explained by variation in amount of huddling and grooming behavior. In the black-penciled marmoset, a closely related species, intranasal oxytocin increased huddling with their partners whereas an oxytocin antagonist decreased proximity, huddling and food sharing [3].

As with oxytocin, prolactin appears to play an important role in the parental care of fathers as well as in mothers in species with biparental or cooperative care. Several studies in rats [4] support the role of prolactin in initiation of maternal care and, in birds, incubation, brooding and feeding of chicks appear to be related to elevations in prolactin in both sexes in species with biparental or cooperative care [5–7].

In mammals several studies have reported elevated prolactin levels in males prior to caring for infants (striped mice [8] meerkats [9] dwarf hamsters [10] cotton top tamarins [11] men [12–15]. In other species the presence of infants appears important for elevated prolactin (California mice [16] Mongolian gerbils [17] common marmosets [18] titi monkeys and Goeldi’s monkeys [19]). Studies in common marmosets have shown that prolactin levels are higher when measured immediately after infant carrying in both breeding males and non-breeding helpers [20–22].

Few studies have experimentally tested the causal effects of prolactin on infant care behavior in male mammals. When the dopamine D_2_ receptor agonist, bromocriptine, was administered to parentally inexperienced male and female juvenile marmosets, prolactin levels were reduced and infant retrieval and caretaking behaviors were also reduced [23]. However, another study using the dopamine D_2_ receptor agonist, cabergoline, which also reduced prolactin levels, reported no effect on infant carrying in paternally experienced marmosets although adult male marmosets did show an increased interest in obtaining proximity to and contact with infants [24]. In common marmosets prolactin levels were decreased through cabergoline administration and chronically elevated using an osmotic mini-pump [25]. Despite the fact that the manipulations significantly affected prolactin levels, there was no difference in infant directed behavior or infant carrying within the family group. However, there was a decrease in responsiveness to infant stimuli to both increased and decreased levels of prolactin using a test of fathers separated from the family group. Similarly there were no effects on infant care in male dwarf hamsters of reducing prolactin levels using either bromocriptine or cabergoline on infant care [26] leading researchers to question the causal role of prolactin in infant care in male mammals [27].

Although the causal role of prolactin in paternal or alloparental care is unclear, there is another possible interpretation of these results, namely that prolactin may be a consequence of infant care and may serve a potentially rewarding function to caregivers. This would explain why prolactin levels are elevated immediately after infant carrying in marmosets and other species as well as explain the lack of effect of prolactin inhibitors on direct infant care. The increased interest in being in physical contact with infants shown in fathers with cabergoline could be interpreted as male seeking greater infant contact to make up for lowered prolactin [24].

There is a relationship between prolactin and physical contact with increased grooming in rats resulting from low doses of prolactin [28–29]. It has been suggested that the continued high prolactin levels in female, but not male, red-cockaded woodpeckers is due to increased tactile stimulation with mothers spending more time in direct contact with chicks [7]. Intracerebral and intraperitoneal injections of prolactin acted centrally to increase levels of courtship behavior in newts [30]. A series of studies on hormones released at orgasm in humans [31–32] demonstrated increased prolactin at orgasm in men and women during both coital sex and masturbation. Thus prolactin appears to play a role in grooming, courtship, and sexual behavior in a variety of species.

Berridge and colleagues [33–35] have distinguished between “wanting” (the desire to obtain something) and “liking” (the positive valence of reaching a goal) with dopamine serving as a critical neurotransmitter during the “wanting” phase. However, other mechanisms must serve to provide a reward (and reduce the “wanting”). Given the reciprocal relationship between prolactin and dopamine [36], we hypothesize that prolactin might be involved in rewarding parents and alloparents for infant care and that grooming and sexual behavior may serve to increase prolactin and provide a reward for adult pair relationships as well.

In this paper we examine the relationship between prolactin and measures of contact affiliation and sexual behavior. We predicted that there would be a positive relationship between both contact affiliation and sexual behavior and chronic levels of prolactin in cotton-top tamarins. We also predicted that there would be a close correlation of prolactin levels between males and females within each pair. We completed two studies. Study 1 compared behavior and hormone levels in current mothers (no longer nursing) with former mothers and females who had never been mothers. The second study examined non-breeding male-female pairs.

## Methods

### Ethics Statement and Animal Care

Cotton-top tamarins are an endangered species and we designed our research to be non-invasive. All animals were born in the colony and were reared by their parents. They were socially housed as pairs or family groups and were housed in large enclosures measuring 1.5 x 0.9 x 2.3 m for pairs and 3.0 x 1.8 x 2.3 m for families. Since tamarins weigh about 1% of an average human, the enclosures were the equivalent of 135 m^2^ living area for pairs and 540 m^2^ area for family groups. The enclosures were constructed of black polyurethane coated steel mesh on anodized aluminum frames. The upper half of the cage was equipped with a series of wooden planks, natural branches, sisal ropes and stainless steel and aluminum platforms. The wooden and rope structures were changed every two to three months for sanitation and to create novel pathways for sensorimotor enrichment. The large cages, social housing, and frequent reconfiguration of arboreal travel routes were considered appropriate enrichment by University veterinarians and the Institutional Animal Care and Use Committee. See also S1 ARRIVE Checklist.

Tamarins received full spectrum overhead light from 0800 to 2000 and ambient temperature was maintained within the range of 25.6–27.8 C^0^. Animals had access to water ad libitum and were fed three times daily on platforms located at least 1 m above the enclosure floor. Yogurt applesauce and supplemental vitamins were provided between 0800 and 0930 shortly after the animals awoke. A main feed was presented between 1145 and 1300 and consisted of Zupreem Marmoset Diet and Purina New World Monkey Chow coated with powdered L-ascorbic acid dissolved in water and topped with supplemental fruits, vegetables, potatoes or bread. Between 1430 and 1700 animals received supplemental protein such as peanuts, hard-boiled egg, cottage cheese, canned tuna or mealworms. Tamarins were never food deprived and were rarely handled except when necessary for veterinary care. Further details of animal husbandry have been described previously [37].

The cages are large enough for humans to enter and collect urine sample non-invasively (see below) without handling the animals. The animals were well habituated to this and showed little response to humans collecting samples. All behavioral observations were made from outside the cage by observers to whom the monkeys were well habituated. The protocol was approved by University of Wisconsin, College of Letters and Science Institutional Animal Care and Use Committee (L00030) and conforms to ASAB/ABS Guidelines for the Use of Animals in Research.

### Subjects

#### Study 1

We studied 11 cotton-top tamarin females. To assure maximum variation, we selected females from three reproductive conditions: four females had never been pregnant and were living in male-female pairs where either the male or female had been sterilized for management purposes; four females were previous mothers (having given birth to between 1 and 11 infants) but were now paired with a sterilized male; three females were currently reproducing with a range of six to 25 previous births) and were sampled after the most recent infants had been weaned so that prolactin levels would not be elevated due to nursing.

#### Study 2

We wanted to study a more socially homogeneous sample, to determine whether prolactin is related to affiliation in males and to see if male and female levels were correlated. We studied 8 pairs where either the male or female had been sterilized for management purposes. We chose to study non-breeding pairs in order to avoid the potential extraneous variance that might arise from the presence of infants in a group. Two of the females had participated in Study 1 a year earlier, but were sampled a second time in order to include simultaneous data from their mate. Limitations on the number of non-breeding pairs in our colony required the re-sampling of these two females.

### Data Collection and Assays

We collected first morning void urine samples from each female in Study 1 and from each male and female in Study 2 twice a week for 4 weeks to cover a complete ovarian cycle (ovarian cycle length ranges from 21–25 days). Lights were turned on at approximately 0800 and an experimenter entered the enclosure holding a plastic container underneath the targeted animal. Animals usually urinated within a few minutes. All monkeys in the colony had experienced such urine collection for several years and showed no signs of disturbance or anxiety. Once the urine sample was collected, it was centrifuged to remove any debris, aliquotted into tubes to which a 0.52M glycerol solution was added and then frozen at −20°C until assayed. We deliberately chose to use urine collection rather than serum sampling to maintain our non-invasive methods, as well as to be able to measure normal behavior between mates without the trauma of capture and blood draws multiple times each week. In addition protein hormones are released in pulses into serum and an overnight accumulation of urine averages across these pulses to provide a more stable measure [38].

We also collected 20 min behavioral observations three times a week during the same four weeks of urine collection with observations alternated between the morning and afternoon. Behavioral categories were based on our previous studies [2, 37]. We collected and summarized social behavior under three broad categories. Contact affiliation behavior included time spent in grooming and huddling. A proximity score (1 = in contact, 2 = within arm’s length, 3 = beyond arm’s length) was taken once each minute and averaged. Sexual interactions included anogenital sniffs, stretches, presentations, erections, complete and incomplete mounts, tongue flicks and head shakes in study 1 and complete and incomplete mounts in Study 2. Scent marks, which are mainly done by females, were summed separately in Study 1 and not used in Study 2 where male and female behavior was being compared. Data were summed over all observations and divided by the number of observation sessions to obtain a mean frequency or duration per session.

Prolactin was assayed using a radio-immunoassay that has been described previously [39]. Urine samples were concentrated 20-fold using centrifuge concentrating tubes that allowed the protein component to collect at the bottom while lower molecular weight components were filtered through a membrane into the supernatant. One ml samples of urine in duplicate were pipetted into Cenricon-10 tubes (10,000 MW cut-off) Amicon, Beverly, MA). The tubes were centrifuged at 5000g in a fixed-angle rotor at 15°C for 100 min. These samples, concentrated to 50 ul were mixed with 250 ul of assay buffer (0.017M sodium phosphate, 0.003M potassium phosphate, 0.5% BSA, 9% sodium chloride, 0.02% sodium azide, pH 7.4) and inverted over 12 x 75 mm glass tubes. The liquid was collected into the bottom of the tube by centrifuging at low speed for 2 min.

The assay was performed by using a human prolactin ^125^I liquid phase double antibody RIA. Purified human prolactin (UCB-Bioproducts, Accurate) was used as the standard in doses ranging from 0.1 to 33.3 ng per tube [39]. The assay has been validated for tamarin urine and biological validations for tamarin prolactin measurement by the human prolactin assay have confirmed the assay. Intra and interassay coefficients of variation for this data set were 8.5% and 9.7% respectively (N = 3).

Creatinine concentrations, using a modified Jaffe method [40], were also performed on each sample and urinary prolactin values were divided by creatinine to correct for differences in urine concentration.

### Data Analyses

Prolactin values for each animal were averaged over all samples collected during the four weeks. In study 1 we used the mean values for sexual behavior (without scent marking) scent marking, huddling and grooming and proximity for each reproductive condition (never mothers, former mothers and current mothers) and tested between conditions using Kruskal–Wallis ANOVAs. There were no differences between females who had never been mothers and former mothers (*p’s* >0.10), and we combined these groups to compare them with current mothers using Mann-Whitney U-tests. Subsequently we computed Spearman Rank Order correlations between prolactin levels, sexual behavior, huddling and grooming, proximity and scent marking. In study 2 we used Spearman Rank Order Correlations to compare prolactin levels and behavior between males and females and compared prolactin levels of each sex with time in contact affiliation (huddling and grooming) and frequency of mounts. All tests were two-tailed with *p* < 0.05. We used SPSS 17.0 for all analyses. Data are presented in S1 Dataset.

## Results

### Study 1

There were significant differences between conditions for sexual behavior (*Х* ^*2*^_(2)_ = 6.42, *p* = 0.040) and for proximity (*Х* ^*2*^_(2)_ = 6.05, *p* = 0.049). Mann Whitney U tests indicated that current mothers engaged in significantly less sexual behavior than other females (mean ± s.e.m. current mothers: 2.69 ± 0.92 acts/session; Non-mothers: 6.23 ± 0.89 acts/ session; *U* = 1, *N’s* 3.8; *p* = 0.024) and were less often in proximity to their partners than other females (mean ± s.e.m., current mothers: 2.79 ± 0.04, non-mothers: 2.29 ± 0.06; *U* = 0, *N’s* = 3, 8, *p* = 0.012). There was a non-significant trend for prolactin levels to be lower for current mothers than for other females (*U* = 3; *N’s* = 3.8; *p* = 0.085).

Prolactin levels were significantly correlated with sexual behavior (*R*_*S*_ = 0.679, *N* = 11, *p* = 0.022) and with scent marking (*R*_*S*_ = 0.742, *N* = 11, *p* = 0.009) with non-significant trends with duration of contact affiliation (*R*_*S*_ = 0.579, *N* = 11, *p* = 0.062) and a negative non-significant trend with proximity (*R*_*S*_ = -0.574, *N* = 11, *p* = 0.065).

### Study 2

**Fig 1:**
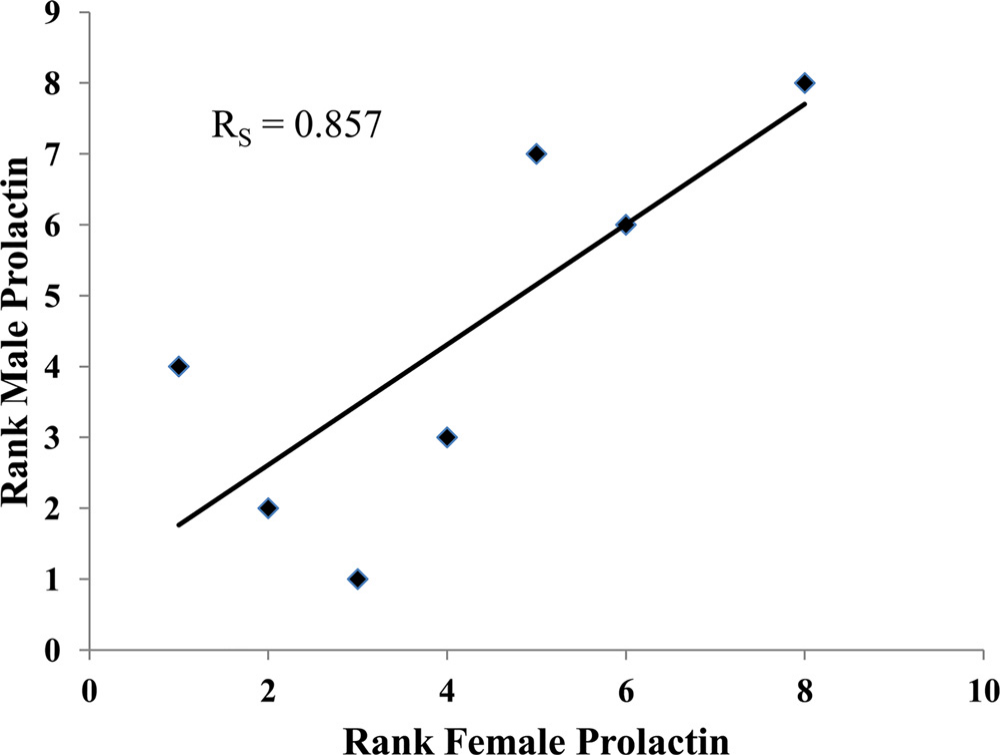
Male-Female Correlation. The correlation between rank prolactin levels in male and female pair-bonded tamarins in Study 2.

**Fig 2:**
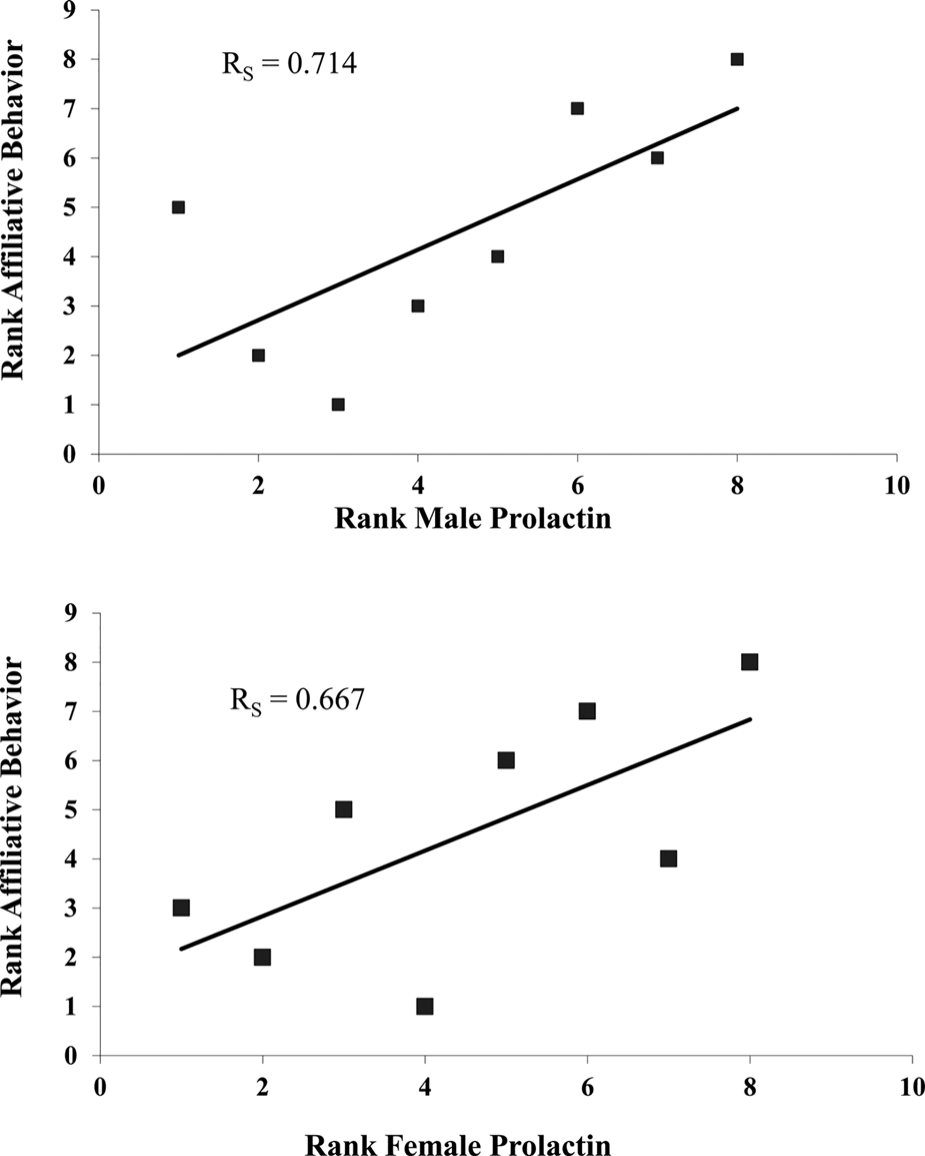
Affiliation and Prolactin. The correlation between rank contact affiliation behavior and rank prolactin levels in Study 2 for males (top) and females (bottom).

Males and females within a pair showed a significant correlation in prolactin levels (*R*_*S*_ = 0.738, *N* = 8, *p* = 0.037, Fig. 1). Male prolactin levels correlated significantly with the amount of sexual behavior (*R*_*S*_ = 0.756, *N* = 8, *p* = 0.030) and with contact affiliation (*R*_*S*_ = 0.714, *N* = 8, *p* = 0.047, Fig. 2) and female prolactin showed a non-significant trend with the duration of contact affiliation (*R*_*S*_ = 0.667, *N* = 8, *p* = 0.07) as was seen in Study 1 as well. However, when we combined data from females of both groups, there was a significant correlation between prolactin and contact affiliation (*R*_*S*_ = 0.578, *N* = 19, *p* = 0.01). See also S1 Dataset.

## Discussion

These are the first results relating urinary prolactin levels in adults with measures of mate affiliation and sexual behavior, and parallel previous results with urinary oxytocin in cotton-top tamarins [2]. Within pairs prolactin levels of males and females were correlated and variation in prolactin levels was explained by variation in amount of sexual behavior (for females in study 1 and males in study 2). Contact affiliation showed a trend toward a significant correlation with female prolactin levels in each study. When both samples were combined, the correlation was significant. In study 2 contact affiliation also was significantly correlated with male prolactin. Thus, chronic peripheral levels of prolactin correlated significantly with both sexual behavior and contact affiliation in both sexes (although the correlation of contact affiliation and prolactin was weaker for females than for males and emerged only when the data from the two studies were combined). This differs slightly from our previous study where variation in male oxytocin was best explained by sexual behavior and variation in female behavior by variation in contact affiliation. Overall however, both prolactin and oxytocin were related to the amount of sexual and contact affiliation behavior.

We were initially puzzled by the lower prolactin levels in current mothers, but the mates of these females are actively involved in infant care and current mothers were significantly less often in proximity with their partners and engaged in less sexual behavior than non-mothers did with their mates.

Although this study was correlational, the findings of increased prolactin after orgasm in humans [31–32] and of elevated prolactin during infant carrying in marmosets [20–22] along with the suggestion that tactile stimulation from brooding of chicks by parents may be related to increased prolactin [7] taken together suggest that increased prolactin levels are a result of sexual behavior and contact affiliation. The increased interest in maintaining contact with infants shown by male marmosets with cabergoline injections [24] is consistent with males seeking infant care to maintain prolactin levels. These results generally support the idea that prolactin may be acting as a reward for parenting as well as for social sexual interactions with a partner. This would suggest that tamarin pairs with higher levels of prolactin should also have a stronger pair bond than those with lower levels.

Berridge and colleagues [35] illustrated an overlap between “wanting” and “liking” functions in the nucleus accumbens and ventral pallidum. They proposed that the main function of dopaminergic systems is to motivate behavior toward a goal whereas opioid or GABA systems serve as rewards or the “liking” system. Given the reciprocal relationships between prolactin and dopamine (with D_2_ receptor agonists inhibiting prolactin) and the fact that GABA and opioids increase hypothalamic secretion of prolactin [36], prolactin may also be important to the “liking” system. Supporting this is the extensive prolactin receptor immunoreactivity in the ventral pallidum and globus pallidus [41]. A study of marmoset hypothalamic explants found a trade-off between dopamine and prolactin. Explants from experienced fathers showed higher levels of prolactin and lower levels of dopamine than males who had never been fathers [42]. The fathers in their study had not had contact with infants for several months suggesting that engaging in parental care may have induced permanent changes in hypothalamic prolactin secretion. Intracerebral prolactin infusions in virgin female rats led to increased oxytocin synthesis and increased secretory activity of both oxytocin and arginine vasopressin neurons [43]. Not only does prolactin stimulate the “liking” system and reduce dopamine activity, prolactin increases oxytocin and vasopressin, which are also involved in pair bonding.

Breeding pairs of tamarins and marmosets engage in high rates of grooming behavior (up to 20% of observations on wild common marmosets, [44]) and we have reported high rates of nonconceptive sex across the ovarian cycle and during pregnancy and have found increased rates of nonconceptive sex and grooming in response to perturbations of the pair relationship in cotton-top tamarins [45]. These behaviors may serve to reinforce the pair relationship likely through the reward mechanisms of increased prolactin and oxytocin. However, we have found little in the literature that has examined the potential role of prolactin as a social reward or as a mechanism to form and sustain a pairbond. Although our sample size is limited, there has been little research on the potential role of prolactin in heterosexual pair bonds. The current results are suggestive that prolactin could be a social reward for pairbonded species, but further research is needed on other pairbonded species and with formal evaluations of the reward value of prolactin in order to support these suggestions.

## Supporting Information

S1 Dataset Prolactin and behavioral data for the subjects in Study 1 and Study 2. (XLSX)

S1 ARRIVE Checklist (PDF)
